# Supplementary material for: Is Piezocision effective in accelerating orthodontic tooth movement: A systematic review and meta-analysis
Source: PLoS One. 2020 Apr 22;15(4):e0231492. doi: 10.1371/journal.pone.0231492 (PMC7176130; doi:10.1371/journal.pone.0231492)
Supplement: S1 Table — (DOCX) [file pone.0231492.s002.docx]

Supplemental

| Pubmed | (((((((Tooth movement) AND Accelerat*)) OR ((Orthodontic*) AND corticision)) OR ((Orthodontic*) AND Piezotome)) OR ((Orthodontic*) AND piezocision)) OR ((((ORTHODONTIC OR ORTHODONTICALLY OR ORTHODONTICS))) AND ((PIEZOCISION OR PIEZOELECTRIC)))) OR (((ACCELERATE OR ACCELERATED OR ACCELERATES OR ACCELERATING) AND (ORTHODONTIC OR ORTHODONTICALLY OR ORTHODONTICS)))" |
| --- | --- |
| Scopus | ( ( ( ( ( ( ( ( tooth  AND movement )  AND  accelerat* ) )  OR  ( ( orthodontic* )  AND  corticision ) )  OR  ( ( orthodontic* )  AND  piezotome ) )  OR  ( ( orthodontic* )  AND  piezocision ) )  OR  ( ( ( orthodontic  OR  orthodontically  OR  orthodontics )  AND  ( piezocision  OR  piezoelectric ) ) ) )  OR  ( ( ( accelerate  OR  accelerated  OR  accelerates  OR  accelerating )  AND  ( orthodontic  OR  orthodontically  OR  orthodontics ) ) ) )  AND  ( LIMIT-TO ( DOCTYPE ,  "ar" ) )  AND  ( LIMIT-TO ( SUBJAREA ,  "DENT" ) )  AND  ( LIMIT-TO ( EXACTKEYWORD ,  "Human" ) )  AND  ( LIMIT-TO ( SRCTYPE ,  "j" ) ) |
| Cochrane | (Acceleration AND orthodontic) OR (Acceleration AND Tooth movement) OR(Orthodontic AND Pizotome)OR(Orthodontic AND Pizocision) OR(Orthodontic AND Pizoelectric)OR(Orthodontic AND cortication)OR(Tooth movement AND cortication)OR (((Tooth movement) AND Accelerat*):ti,ab,kw OR ((Orthodontic*) AND corticision):ti,ab,kw OR ((Orthodontic*) AND Piezotome):ti,ab,kw OR (((Orthodontic*) AND piezocision):ti,ab,kw OR ((((ORTHODONTIC OR ORTHODONTICALLY OR ORTHODONTICS) AND (PIEZOCISION OR PIEZOELECTRIC))))):ti,ab,kw) |
