# Supplementary material for: Is Piezocision effective in accelerating orthodontic tooth movement: A systematic review and meta-analysis
Source: PLoS One. 2020 Apr 22;15(4):e0231492. doi: 10.1371/journal.pone.0231492 (PMC7176130; doi:10.1371/journal.pone.0231492)
Supplement: S2 Table — (DOCX) [file pone.0231492.s003.docx]

| 1 | Yu 2013 | Piezoelectric Decortication Applied in Periodontally Accelerated Osteogenic Orthodontics | No comparison group |
| --- | --- | --- | --- |
| 2 | Vercellotti 2007 | Orthodontic Microsurgery:  A New Surgically Guided Technique for Dental Movement | Case series |
| 3 | Khanna 2014 | Evaluation of canine retraction following periodontal distraction using NiTi coil spring and implants e A clinical study | They used tapered tungsten carbide bur |
| 4 | Shoreibah 2012 | Corticotomy-facilitated Orthodontics in Adults Using a Further  Modified Technique | They used small round stainless steel surgical bur |
| 5 | Jahanbakhshi 2016 | The effect of buccal corticotomy on accelerating orthodontic tooth movement of maxillary canine | They used surgical bur |
| 6 | CASSETTA 2012 | The impact of osteotomy technique for corticotomy-assisted orthodontic treatment (CAOT) on oral health-related quality of life | Comparison group is non-conventional orthodontic |
| 7 | Bhattacharya 2014 | Assessment of Corticotomy Facilitated Tooth Movement and Changes in Alveolar Bone Thickness - A CT Scan Study | They used surgical bur |
| 8 | Al-Naoum 2013 | Does alveolar corticotomy accelerate orthodontic tooth movement when retracting upper canines? A split-mouth-design randomized controlled trial | They used a fissure bur |
| 9 | Aboul-Ela 2011 | Miniscrew implant-supported maxillary canine retraction with and without corticotomy-facilitated orthodontics | corticotomy perforations |
| 10 | Fischer 2007 | Orthodontic Treatment Acceleration with Corticotomy-assisted Exposure of Palatally Impacted Canines | Small sample size of 6 patients, also they used bur to do holes |
| 11 | Khalid h Zawawi | Patients’ acceptance of corticotomy-assisted orthodontics | Different outcomes  study design |
| 12 | Charavet 2018 | Selective piezocision-assisted orthodontic treatment combined with minimally invasive alveolar bone regeneration:  A proof-of-concept | Different outcome study design |
| 13 | Nimeri 2013 | Acceleration of tooth movement during orthodontic treatment - a frontier in Orthodontics | Different outcome and study design |
