# Supplementary material for: Is Piezocision effective in accelerating orthodontic tooth movement: A systematic review and meta-analysis
Source: PLoS One. 2020 Apr 22;15(4):e0231492. doi: 10.1371/journal.pone.0231492 (PMC7176130; doi:10.1371/journal.pone.0231492)
Supplement: S3 Table — (DOCX) [file pone.0231492.s004.docx]

Risk of bias assessment according to ROB2 tool.

| Study | Randomization process | Deviations from intended | Missing outcome data | Measurement of the outcome | Selection of the reported result | Overall Bias |
| --- | --- | --- | --- | --- | --- | --- |
| Uribe | Some concerns  The baseline differences between intervention and control groups suggest a problem with the randomization process as  female ratio in groups was: 7: 10 and Irregularity index at T0 was 8.32: 6.73 | High risk  No mention of blinding of the patients and the operators.  The authors did not do intention-to-treat (ITT) analyses and this may have substantial impact on the result. | High risk  The results can be biased by the missing data.  Allocated patients were 19: 16, but the analyzed patients were  16:13. | Low risk  The measurement method is appropriate | Low risk  all reported results for the outcome domain correspond to all intended outcome measurements. | High risk  high risk of bias in at least one domain |
| Tuncer | Low risk  The allocation sequence was random and adequately concealed. Also. There was no information about baseline imbalances | Some concerns  Blinding of either the investigator or patients was not possible, and the deviations arise because of the experimental context.  Also, the authors did not mention ITT analysis that can be used to estimate the effect of assignment to intervention. | Low risk  Outcome data were available for all, or nearly all, randomized participants | High risk  The authors reported that all measurements were done in the patients’ mouth and for right and left sides separately. This method has poor validity. | Low risk  all reported results for the outcome domain correspond to all intended outcome measurements. | High risk  high risk of bias in at least one domain |
| Abbas | Some concerns  There is no information about allocation concealment. Also, there is no baseline characteristic for the intervention side and the control side. | High risk  There is no information about the blinding of the patients and the operator, and it may be possible they know the intervention side and the control side. Also, there is no information regarding the deviations from intended interventions. | Low risk  Outcome data were available for all, or nearly all, randomized participants | Some concerns  Although the measurement method is appropriate, there is no information if the outcome assessors were aware of the intervention received (the scars) which may affect the outcome especially periodontal indexes. | Low risk  all reported results for the outcome domain correspond to all intended outcome measurements. | High risk  high risk of bias in at least one domain |
| Aksakali | Some concerns  There is no information regarding allocation concealment. | Some Concerns  There was no blinding for the patients and the operator, with no information regarding deviations from the intended intervention. | Low risk  Outcome data were available for all, or nearly all, randomized participants | Low risk  The measurement method is appropriate | Low risk  all reported results for the outcome domain correspond to all intended outcome measurements. | Some Concerns  some concerns in at least one domain |
| Alfawal | Low risk  The allocation sequence was random and adequately concealed. Also. There was no information about baseline imbalances | Some Concerns  There is missing data in the both groups without ITT analysis. | Low risk  Outcome data were available for all, or nearly all, randomized participants | Low risk  The measurement method is appropriate | Low risk  all reported results for the outcome domain correspond to all intended outcome measurements. | Some concerns  some concerns in at least one domain |
| Charavet 2016 | Some Concerns  There is no information regarding the allocation concealment. Also, the difference in the age between groups suggests a problem in the randomization process.  The mean age was 27 years in the control group vs 34 years in the piezocision group. | Some Concerns  There was no blinding for the patients and the operator, with no information regarding deviations from the intended intervention. | Low risk  Outcome data were available for all, or nearly all, randomized participants | High Risk  During treatment, the change of the archwires is done by operator thus he recognizes the piezocision patients and this may affect the outcome. | Low risk  all reported results for the outcome domain correspond to all intended outcome measurements. | Some Concerns  some concerns in at least one domain |
| Charavet 2019 | Low risk  The allocation sequence was random and adequately concealed. Also. There was no information about baseline imbalances | Low risk  Patients and operators were aware of intervention groups during the trial but there is no deviations from intended intervention arose because of the experimental context | Low risk  Outcome data were available for all, or nearly all, randomized participants | Low risk  The measurement method is appropriate | Low risk  all reported results for the outcome domain correspond to all intended outcome measurements. | Low risk  low risk of bias for all domains |
| Charavet (PROMs) 2019 | Low risk  The allocation sequence was random and adequately concealed. Also. There was no information about baseline imbalances | Low risk  Patients and operators were aware of intervention groups during the trial but there are no deviations from intended intervention arose because of the experimental context | Low risk  Outcome data were available for all, or nearly all, randomized participants | High risk  There is a deference in the time points for groups, the control is assessed for 7 days after appliance bonding and the intervention group is assessed 7 days after bonding and also after additional week, with more visits for the intervention group. Also, the patients were assessor of the pain, and as they know the surgical procedure, this may create bias toward pain assessment. (the assessor is aware of the intervention) | Low risk  all reported results for the outcome domain correspond to all intended outcome measurements. | High risk  high risk of bias in at least one domain |
| Gibreal 2018 | Low risk  The allocation sequence was random and adequately concealed. Also. There was no information about baseline imbalances | Some Concerns  There is missing data in the both groups without ITT analysis. | Low risk  Outcome data were available for all, or nearly all, randomized participants | Low risk  The measurement method is appropriate | High risk  The authors have two different outcomes (one for decrowding and one for the duration of alignment)  They reported the decrwoding take more than two months to be resolved in piezocision group. In contrast they reported that the alignment has done by less than two months in piezocision group. | Some Concerns  some concerns in at least one domain |
| Gibreal 2019 | Low risk  The allocation sequence was random and adequately concealed. Also. There was no information about baseline imbalances | Some Concerns  There is missing data in the both groups without ITT analysis. | Low risk  Outcome data were available for all, or nearly all, randomized participants | High risk  There is a deference in the time points for groups. The time point for recording the outcome is not clear for the two groups, as this point was at specific days following the onset of treatment, but there is no clear relation with the intervention and this mean measurement differences between groups. | Low risk  all reported results for the outcome domain correspond to all intended outcome measurements. | High risk  high risk of bias in at least one domain |
| Al-Imam 2019 | Low risk  The allocation sequence was random and adequately concealed. Also. There was no information about baseline imbalances | Some Concerns  Blinding of either the investigator or patients was not possible, and the deviations arise because of the experimental context. | Low risk  Outcome data were available for all, or nearly all, randomized participants | Low risk  The measurement method is appropriate | Low risk  all reported results for the outcome domain correspond to all intended outcome measurements. | Some Concerns  some concerns in at least one domain |
| Raj 2020 | Some Concerns  There is no information regarding allocation concealment | High risk  There is missing data in the both groups without ITT analysis, and also the excluded data may have impact on the results (more than 20%) | Some Concerns  The availability of data from 95% of the participants would often be sufficient. In this study the availability of data is 80% but the result was not biased by missing outcome data (split mouth design). | High risk  The method of measuring is not appropriate (canine retraction rate was measured by the distance between the mesial aspect of the molar tube slot and the distal aspect of the canine bracket, measured intraorally)  The method of measurement is not valid | Low risk  all reported results for the outcome domain correspond to all intended outcome measurements. | High risk  high risk of bias in at least one  domain |
